# Single-Cell Transcriptional Profile Construction of Rat Pituitary Glands before and after Sexual Maturation and Identification of Novel Marker *Spp1* in Gonadotropes

**DOI:** 10.3390/ijms25094694

**Published:** 2024-04-25

**Authors:** Qing-Hua Huang, Guo-Kun Zhao, Hao-Qi Wang, Fan-Hao Wei, Jin-Yu Zhang, Jia-Bao Zhang, Fei Gao, Bao Yuan

**Affiliations:** Department of Laboratory Animals, College of Animal Sciences, Jilin University, Changchun 130062, China; huangqh20@mails.jlu.edu.cn (Q.-H.H.); zhaogk20@mails.jlu.edu.cn (G.-K.Z.); wanghq21@mails.jlu.edu.cn (H.-Q.W.); weifh22@mails.jlu.edu.cn (F.-H.W.); zjy3685@jlu.edu.cn (J.-Y.Z.); zjb@jlu.edu.cn (J.-B.Z.)

**Keywords:** rat pituitary, single-cell RNA sequencing, puberty, sexual maturity, SPP1

## Abstract

The mammalian pituitary gland drives highly conserved physiological processes such as somatic cell growth, pubertal transformation, fertility, and metabolism by secreting a variety of hormones. Recently, single-cell transcriptomics techniques have been used in pituitary gland research. However, more studies have focused on adult pituitary gland tissues from different species or different sexes, and no research has yet resolved cellular differences in pituitary gland tissue before and after sexual maturation. Here, we identified a total of 15 cell clusters and constructed single-cell transcriptional profiles of rats before and after sexual maturation. Furthermore, focusing on the gonadotrope cluster, 106 genes were found to be differentially expressed before and after sexual maturation. It was verified that *Spp1*, which is specifically expressed in gonadotrope cells, could serve as a novel marker for this cell cluster and has a promotional effect on the synthesis and secretion of follicle-stimulating hormone. The results provide a new resource for further resolving the regulatory mechanism of pituitary gland development and pituitary hormone synthesis and secretion.

## 1. Introduction

The pituitary gland, composed of the adenohypophysis and neurohypophysis regions, responds to signals from hypothalamic neurons and regulates physiological activities such as growth, metabolism, reproduction, and immunity in mammals. Most pituitary hormones are synthesized and secreted by adenohypophysis. It is now generally accepted that the formation of the Rathke pouch represents the onset of adenohypophysis development [1]. This process is dependent on the collaboration of LHX3/4 [2], PITX1/2 [3], SOX2/3 [4], and other transcription factors [5]. Cyst wall cells segregate and progressively form three major endocrine cell lineages, according to the specific expression of various genes. Among these, *Pou1f1*-expressing cells differentiate into somatotropes, lactotropes, and thyrotropes [6,7]; *Tbx19*-expressing cells differentiate into corticotropes and melanotropes [8]; and *Nr5a1*-expressing cells differentiate into gonadotropes [9]. Multiple pituitary hormones secreted by corresponding endocrine cells interact with one another to maintain the normal coordination of physiological activities. It is expected that the further exploration of the molecular differences between different types of endocrine cells will help to better resolve the mechanisms of pituitary hormone synthesis and to gain a deeper understanding of the physiological roles of pituitary hormones.

It has become possible to achieve the fine delineation of pituitary cell types with the continuous development of single-cell transcriptomics technology. Several single-cell-level studies of the pituitary gland have demonstrated that there is a complex sinusoidal capillary network of follicle-like cells, proliferating cells, endothelial cells, peripheral cells, epithelial cells, and mesenchymal cells in the pituitary gland, in addition to hormone-secreting endocrine cells [10,11,12]. Somatotropes, gonadotropes, and corticotropes can also be more finely divided into multiple cellular subpopulations. Polysecretory cells or progenitor cells that simultaneously express marker genes such as *Poulf1*, *Prl*, and *Gh1* have also been progressively identified through the careful analysis of single-cell transcriptomics [13]. The wide application of single-cell transcriptomics has provided new strategies and approaches for the study of pituitary gland development and pituitary hormone secretion.

Puberty, a critical period in the development of the mammalian reproductive system, has always been of great interest for its differential and complex hormone secretion compared to other phases of the lifespan [14]. The changes in sex hormones caused by gonadotropins synthesized and secreted by gonadotropes are the initiating factor and regulatory basis for sexual development during puberty [15]. Large differences in the levels of gonadotropin synthesis and secretion have been seen in mammals at different stages of development. Huayun Hou et al. [16] found that FSH and LH levels in male mice increased gradually during pre-puberty, peaked at approximately 5 weeks of age, and then gradually decreased. However, the molecular mechanisms leading to the reductions in gonadotropin levels in adulthood remain unclear. The related key factors regulating gonadotropin synthesis and secretion at different developmental stages also need to be further explored and investigated. Therefore, in order to further comparatively analyze the changes in heterogeneity and transcriptional dynamics between pituitary cell populations before and after sexual maturation, single-cell transcriptome sequencing was performed on pituitary tissues from 4-week-old (pre-puberty) and 12-week-old (post-sexual maturity) rats in this study. This study aims to explore the differences in gene expression and cell populations in the rat pituitary gland before and after sexual maturation at the single-cell level. The results will provide data to support the elucidation of the potential molecular mechanisms that regulate the reduced level of gonadotropin secretion in rats after sexual maturation.

## 2. Results

### 2.1. Analysis of Pituitary Hormone Secretion Levels before and after Sexual Maturation in Rats

**Figure 1:**
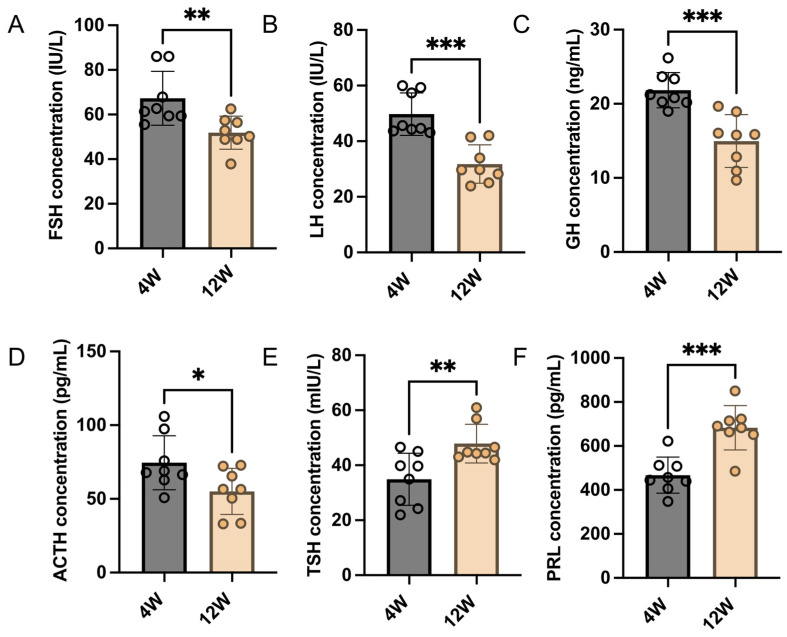
Comparison of pituitary hormone secretion levels in 4- and 12-week-old rats. (**A**) ELISA to detect the secretion of FSH in 4- and 12-week-old rats (n = 8). (**B**) ELISA to detect the secretion of LH in 4- and 12-week-old rats (n = 8). (**C**) ELISA to detect the secretion of GH in 4- and 12-week-old rats (n = 8). (**D**) ELISA to detect the secretion of ACTH in 4- and 12-week-old rats (n = 8). (**E**) ELISA to detect the secretion of TSH in 4- and 12-week-old rats (n = 8). (**F**) ELISA to detect the secretion of PRL in 4- and 12-week-old rats (n = 8). *, *p* < 0.05; **, *p* < 0.01; ***, *p* < 0.001.

To validate the differences in the secretion of pituitary hormones between prepubertal and adult rats, the serum levels of six pituitary hormones were analyzed in 4-week-old and 12-week-old rats. The ELISA results showed that the secreted levels of FSH (Figure 1A), LH (Figure 1B), GH (Figure 1C), and ACTH (Figure 1D) were significantly reduced in adult rats compared to prepubertal rats. Conversely, the secreted levels of TSH (Figure 1E) and PRL (Figure 1F) were significantly increased in adult rats. This suggests that there are differences in the synthesis and secretion of pituitary hormones in rats before and after sexual maturation.

### 2.2. Construction of a Single-Cell Transcriptome Atlas of the Rat Pituitary Gland before and after Sexual Maturation

To explore the pituitary gland differences between the two periods at the single-cell level, single-cell transcriptome sequencing was performed on pituitary tissues from 4-week-old and 12-week-old male rats. The sequencing captured 7807 (4 weeks old, 78% capture efficiency) and 9112 (12 weeks old, 91% capture efficiency) single cells, respectively, with a genome sequence alignment rate of approximately 95% (Supplementary Table S3). The two datasets were used to remove low-quality cells, double cells, mitochondria-contaminated cells, and erythrocytes to obtain a total of 14,530 single cells for subsequent analysis (Supplementary Figure S1A–D).

**Figure 2:**
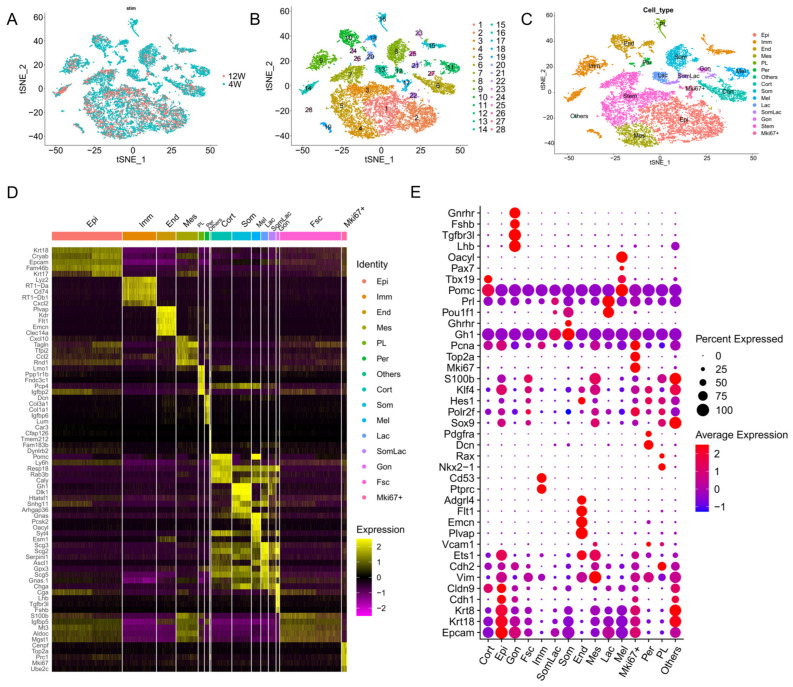
The major cell clusters identified in male rats’ pituitary. (**A**,**B**) UMAP visualization of unintergrated clusters. (**C**) Manual assignment of cells to expected cell types based on gene expression of known markers. (**D**) Heatmap of cluster marker genes. Heatmap shows the top 5 average expressions of cluster marker genes. (**E**) The Dot-plot of the percentage (pct. exp.) and average expression (avg. exp.) of marker genes. Cort, corticotropes; End, endothelial; Epi, epithelial; Gon, gonadotrope; Imm, immune cells; Lac, lactotropes; Mel, melanotropes; Mes, mesenchymal; Mki67+, Mki67+ proliferating cells; Per, pericytes; PL, posterior lobe; Som, somatotropes; SomLac, somatolactotropes; Fsc, folliculostellate cells.

**Figure 3:**
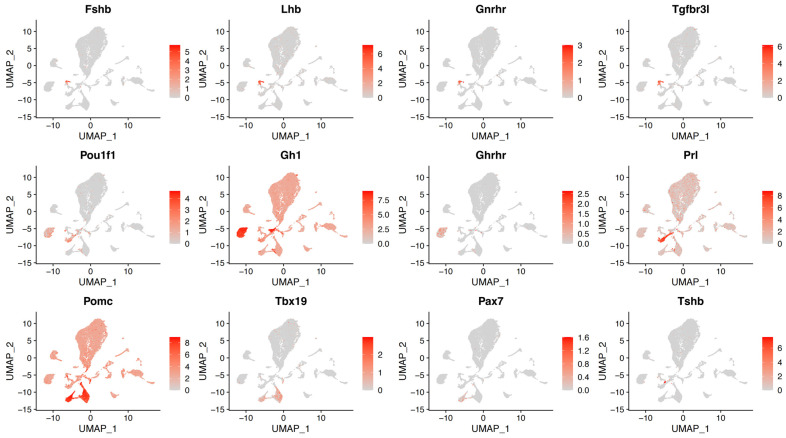
UMAP maps of marker genes in distinct pituitary hormone cell clusters. Classical pituitary endocrine cell markers are all shown by UMAP maps. Color bar indicates natural log transformed normalized expression.

The tSNE plot results were similar for both samples, with no specific groups expressed by a single sample. A total of 28 cell populations were isolated by single-cell transcriptomics (Figure 2A,B). They were further classified into 15 cell clusters (Figure 2C–E) based on specifically expressed marker genes (Supplementary Table S4), including those of epithelial cells (Epi, cluster 1, 2, and 22), endothelial cells (End, cluster 10, 18, and 24), folliculostellate cells (Fsc, cluster 3, 5, and 7), immune cells (Imm, cluster 9, 14, 15, 19, and 23), mesenchymal cells (Mes, cluster 4), pericytes (Per, cluster 20 and 26), somatotropes (Som, cluster 8 and 25), lactotropes (Lac, cluster 13), somatolactotropes (SomLac, cluster 12), gonadotropes (Gon, cluster 21), melanotropes (Mel, cluster 11), corticotropes (Cort, cluster 6 and 27), posterior lobe cells (PL, cluster 16), and Mki67+ cells (Mki67+, cluster 17). In addition, cluster 28 simultaneously expressed lineage-specific transcription factors and hormone-related genes such as *Lhb*, *Sox9*, *S100b*, *Klf4*, *Krt8*, and *Krt18*. It is hypothesized that these cells may have been contaminated and defined as other cells (Others). UMAP maps showed the expressions of the anterior pituitary endocrine markers *Fshb*, *Lhb*, *Gnrhr*, *Tgfbr3l*, *Pou1f1*, *Gh1*, *Ghrhr*, *Prl*, *Pomc*, *Tbx19*, *Pax7*, and *Tshb*. This confirmed that gonadotropes, somatotropes, lactotropes, corticotropes, and melanotropes, respectively, are all detected in our samples (Figure 3). Curiously, no clusters of thyrotropes were formed despite the identification of Tshb expression, possibly due to the loss of cell clusters caused by cell dissociation. The expressions of the anterior pituitary non-hormone markers were also shown by UMAP maps (Supplementary Figure S2).

**Figure 4:**
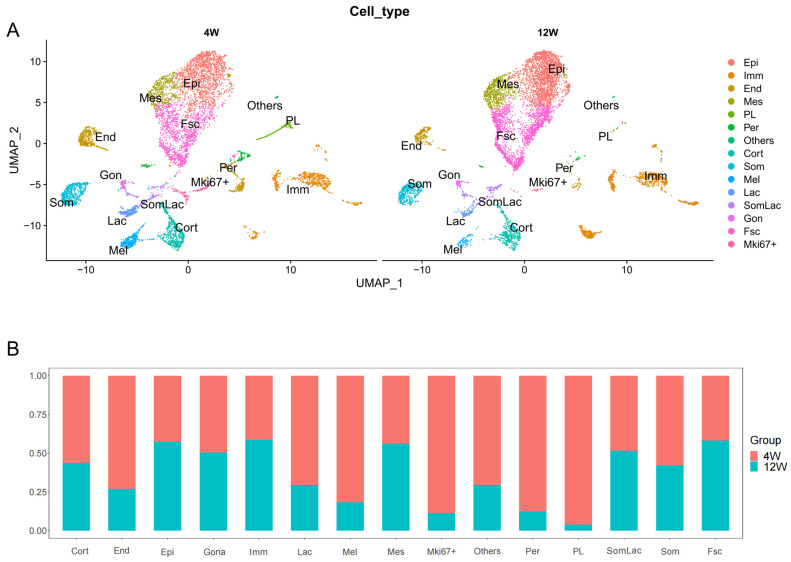
Cell type composition between 4- and 12-week-old male rat pituitary cells. (**A**) UMAP visualization of 4- and 12-week-old male rat pituitary cells. Dots: single cells. (**B**) Bar graph of cell type composition between 4- and 12-week-old male rats.

A comparison of the proportions of rat pituitary cell clusters before and after sexual maturation showed significant differences (Figure 4A,B). In the 12-week-old pituitary glands, the proportions of eight cell clusters decreased compared to those of the 4-week-old rats, including Cort, End, Lac, Mel, Som, Mki67+, Per, and PL. The proportions of four cell clusters increased, including Epi, Imm, Mes, and Fsc. No significant changes were observed in the Gon and SomLac clusters (Supplementary Table S5). Interestingly, despite differences in the overall proportions of some cell clusters, such as Cort, Som, and Fsc, the numbers and proportions of a small subset of these cells did not change dramatically between the two periods. This suggests the need for the further refinement of cell clusters to gain insight into intercellular differences and functions.

### 2.3. Enrichment Analysis of Differentially Expressed Genes in Gonadotrope Cells before and after Sexual Maturation

**Figure 5:**
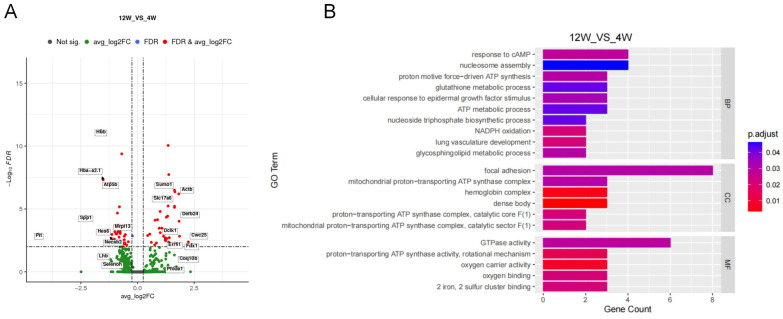
Enrichment analysis of differentially expressed genes in gonadotrope cells before and after sexual maturation. (**A**) Volcanic map of differentially expressed genes in the gonadotrope cell. (**B**) Major enrichment and meaningful GO terms of differentially expressed genes in the gonadotrope cell.

Although secreted levels of gonadotropin decreased significantly in adulthood, gonadotropes did not show significant differences. This suggests that differences in gonadotropin synthesis and secretion may result from the temporal sequence of the expression of certain specific genes. To explore the key factors regulating the synthesis and secretion of gonadotropins, gene expression changes in gonadotrope cells during the period before and after sexual maturation were analyzed. The results showed that a total of 106 genes were differentially expressed, 45 of which were significantly upregulated and 61 of which were significantly downregulated (Figure 5A and Supplementary Table S6). GO enrichment analysis was performed on the differentially expressed genes to examine the functional details (Figure 5B). In the biological process module, the differentially expressed genes were significantly enriched for the glycosphingolipid metabolic process, NADPH oxidation response to cAMP, and cellular response to an epidermal growth factor stimulus. In the cellular component module, the differentially expressed genes were significantly enriched for focal adhesion, a hemoglobin complex, and dense body. In the molecular function module, the differentially expressed genes were significantly enriched for GTPase activity and oxygen carrier activity (Supplementary Table S7).

### 2.4. Validation of Differentially Expressed Genes in Different Cell Types

**Figure 6:**
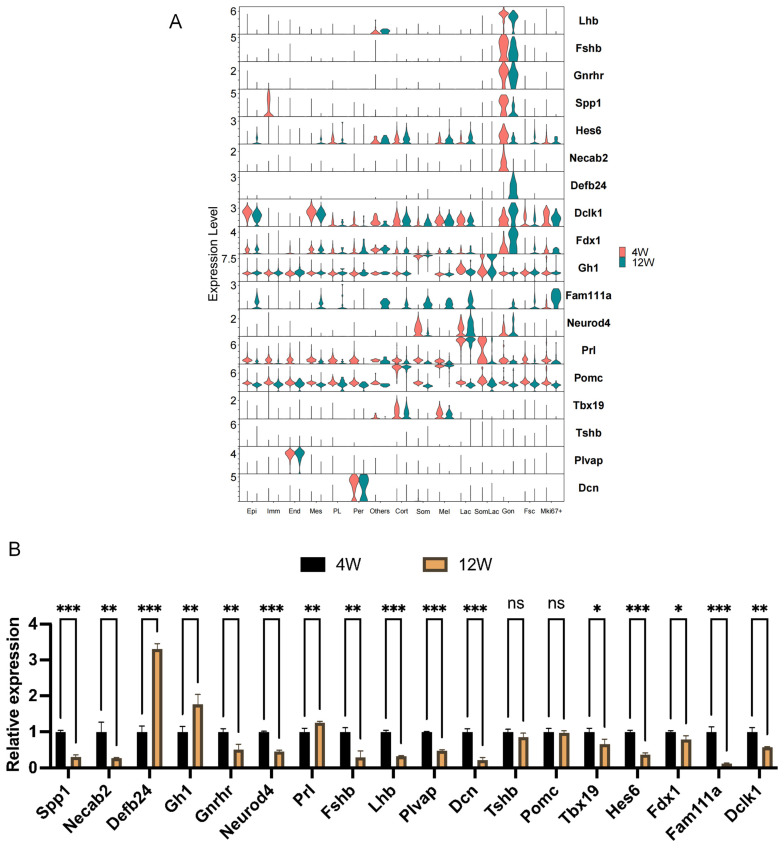
Validation of differentially expressed genes. (**A**) Differential expression of selected genes in sequencing results. The orange column represents pituitary tissue samples of 4-week-old rats, and the green column represents pituitary tissue samples of 12-week-old rats. (**B**) Validation of differentially expressed genes by RT-qPCR. The black column represents pituitary tissue samples of 4-week-old rats, and the yellow column represents pituitary tissue samples of 12-week-old rats. ns, no statistical difference; *, *p* < 0.05; **, *p* < 0.01; ***, *p* < 0.001.

To verify the accuracy of the histological results, 18 genes specifically expressed in different cell clusters (marker genes) or significantly differentially expressed in gonadotrope cells were randomly selected, and their expression levels in the period before and after sexual maturation were examined by RT-qPCR. The RT-qPCR results (Figure 6B) revealed that the expression trends of the genes were all consistent with the results of scRNA-seq (Figure 6A), although the expression changes in very few genes were not as highly significant as was seen in the sequencing results.

### 2.5. Identification and Validation of Spp1: A Novel Marker for Gonadotrope Cell Types’ Mining and Analysis of Novel Markers of Pituitary Hormone-Secreting Cells

**Figure 7:**
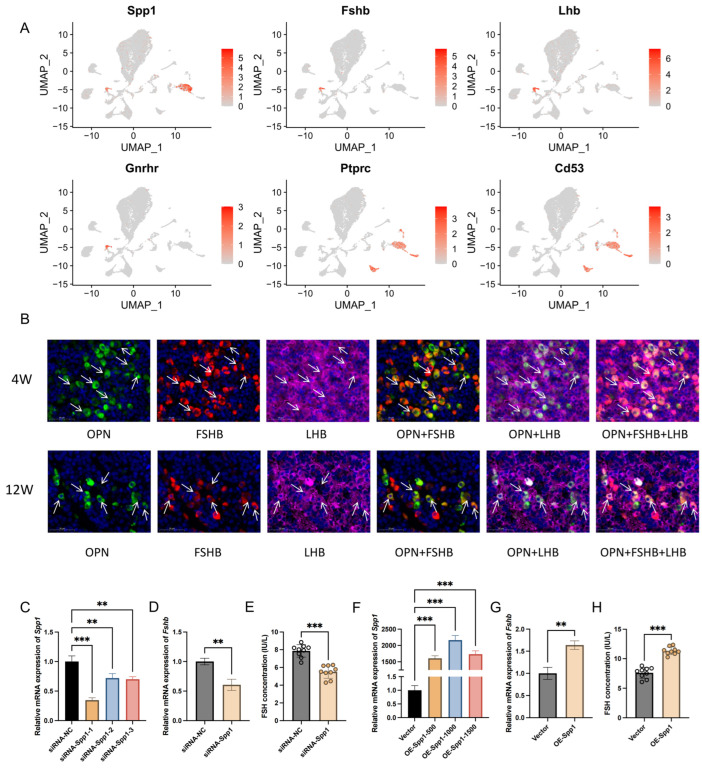
*Spp1* promotes FSH synthesis and secretion. (**A**) Pituitary single-cell transcriptomic analysis of *Spp1* mRNA enrichment in different cell populations. (**B**) Immunofluorescence analysis OPN (green), FSHB (red), and LHB (purple) in pituitary tissue of 4-week-old and 12-week-old rats. The white arrows point to representative co-localization. (**C**) RT-qPCR analysis *Spp1* mRNA expression in LβT2 cells transfected with siNC and three Spp1 siRNAs (n = 3). (**D**) RT-qPCR analysis *Fshb* mRNA expression transfected with siNC and Spp1 siRNA in LβT2 cells (n = 3). (**E**) ELISA analysis secretion level of FSH transfected with siNC and Spp1 siRNA in LβT2 cells (n = 9). (**F**) RT-qPCR analysis *Spp1* mRNA expression in LβT2 cells transfected with vector and different quality of *Spp1* plasmid (n = 3). (**G**) RT-qPCR analysis *Fshb* mRNA expression transfected with vector and *Spp1* plasmid (1000 ng) in LβT2 cells (n = 3). (**H**) ELISA analysis secretion level of FSH transfected with vector and *Spp1* plasmid (1000 ng) in LβT2 cells (n = 9). **, *p* < 0.01; ***, *p* < 0.001. Cort, corticotropes; End, endothelial; Epi, epithelial; Gon, gonadotrope; Imm, immune cells; Lac, lactotropes; Mel, melanotropes; Mes, mesenchymal; Mki67+, Mki67+ proliferating cells; Per, pericytes; PL, posterior lobe; Som, somatotropes; SomLac, somatolactotropes; Fsc, folliculostellate cells; Others, other cells.

On the basis of the validation of classic cell marker genes, new markers for clusters of hormone-secreting cells were also identified (Supplementary Table S8). The scRNA-seq observations were validated by in vitro studies to demonstrate the reliability of the scRNA-seq technique for identifying new cluster markers. UMAP maps of single-cell transcriptomics results showed that the *Spp1* gene was more specifically expressed in gonadotrope cells, although it was also expressed in macrophages (Figure 7A). The immunofluorescence results confirmed that the protein OPN encoded by the *Spp1* gene was specifically enriched in gonadotrope cells (Figure 7B). These results suggest that *Spp1* is capable of being a marker to mark the gonadotrope cell cluster.

To investigate the potential function of the *Spp1* gene, which is specifically expressed in gonadotrope cells and is significantly differentially expressed before and after sexual maturation, a siRNA and a plasmid of *Spp1* were sequentially transfected into LβT2 cells. The RT-qPCR results showed that the silencing of *Spp1* significantly reduced the expression of *Fshb* mRNA (Figure 7D). An analysis of FSH secretion by ELISA also showed that the silencing of *Spp1* significantly inhibited the FSH secretion level (Figure 7E). In contrast, the overexpression of *Spp1* showed the opposite result (Figure 7G,H). The above results indicate that *Spp1* has a promotional effect on FSH synthesis and secretion. This may be one of the mechanisms regulating FSH synthesis and secretion before and after sexual maturation.

## 3. Discussion

Fluctuations in the levels of pituitary hormone secretion affect mammalian development and reproductive activity. It is vital to clarify the regulatory mechanisms of pituitary hormone differences for the systematic resolution of differences in pituitary cell types, ratios, and key factors before and after sexual maturation. In this study, we analyzed the differences in different types of cells in the rat pituitary gland before and after sexual maturation based on single-cell transcriptome technology, which helps to better dissect the intrinsic mechanisms underlying the differences in hormone secretion during different developmental periods in mammals.

In this study, we first examined the serum hormone levels in pre-pubertal and post-sexual maturity rats and found that secreted levels of FSH, LH, GH, and ACTH in the peripheral blood of post-sexual maturity rats were significantly lower than those of pre-pubertal rats. Conversely, the levels of TSH and PRL were significantly higher in post-sexual maturity rats than those of pre-puberty rats. The cause of this difference in hormone levels may be the result of the regulation of multiple physiologic factors. FSH and LH are regulated by hormones such as GnRH, testosterone, and inhibin [17]. Compared with four-week-old rats, high levels of testosterone in sexually mature rats reduce the frequency of the GnRH pulse release, creating the negative feedback. This may be the main reason for the reduced levels of FSH and LH secretion in sexually mature rats. Reduced levels of GH are more likely to be due to its physiological function of regulating protein synthesis, the glycometabolism, and fat metabolism and promoting growth in mammals [18,19,20]. The need for GH to maintain growth and development is greater during puberty than after sexual maturation. Elevated TSH levels are hypothesized to be associated with increased reproductive activity after sexual maturity. It is widely recognized that thyroxine is associated with a reproductive function [21,22]. A decrease in thyroxine production would result in lower gonadotropin levels and reduced gonadal activity [23,24]. Therefore, the increase in TSH levels after sexual maturity may be to promote the synthesis of thyroxine in order to maintain a reproductive capacity. Serum PRL levels in rats are low during the first three weeks of life and gradually increase as they enter puberty [25]. Estrogen, 5-hydroxytryptamine, TRH, and other prolactin-releasing factors can influence PRL release [26]. The gradual increase in PRL levels in male rats after puberty may be related to the development of the testes and accessory organs [27]. After sexual maturity, PRL regulates patrilineal behavior in male rats and may promote the actions of LH, FSH, and testosterone on the testes [28]. Regarding ACTH, its synthesis is not limited to adenohypophysis, and it has been found that the testes are also capable of producing ACTH [29]. ACTH also has potential functions in spermatogenesis, immunity, and other aspects [30,31,32]. The decreased results in this study are more likely to be an effect of suppressed GnRH stimulation. In the future, more reliable evidence is needed to analyze the molecular mechanisms of these hormone level changes and their regulatory relationships to support the above conjectures.

Furthermore, the single-cell transcriptome sequencing of pituitary glands from both periods identified a total of 15 cell clusters. The results of this pituitary cell identification and clustering are in general agreement with the results of Jie Qiao et al. [13] and other researchers [33,34]. However, the present study did not identify thyrotropes, a cluster of cells clearly present in the pituitary gland. It is hypothesized that the loss of this cell cluster may be due to cell dissociation. This phenomenon of a loss of cell clusters was also noted by Alexandre Mayran et al. [11]. The presence of this phenomenon again suggests that the accuracy of the histologic results needs to be repeatedly verified by more in vivo and in vitro studies.

FSH secreted by gonadotropes regulates mammalian reproduction and development [35]. Genes specifically expressed in gonadotrope cells play an important role in regulating FSH secretion. This study analyzed gene expression changes in gonadotropes before and after sexual maturation and identified 106 differentially expressed genes, including 45 up-regulated genes and 61 down-regulated genes. GO enrichment analysis revealed that the differentially expressed genes were significantly enriched in cAMP- and ATP-related terms. This may be due to the key role of the cAMP/PKA/CREB signaling pathway in regulating FSH synthesis and secretion [17,36]. However, due to the small number of differentially expressed genes identified, this study did not perform the KEGG enrichment analysis, which is one of the limitations of this study.

Novel marker genes are being mined and characterized with the application of single-cell transcriptomics. Leonard Y. M. Cheung et al. [33] performed single-cell transcriptome analysis on the pituitaries of 7-week-old C57BL/6 male mice and found that the classic transcription factor *Foxp2* is specifically expressed in gonadotropes. Similarly, we found in the present study that *Spp1* is specifically expressed in gonadotropes. Although SPP1 has been identified in several previous studies, no studies have validated its accuracy and potential function as a marker gene [37]. Recently, Ivana Bjelobaba et al. [38] analyzed *Spp1* expression during rat development and found that sex differences in *Spp1* expression began to appear at 4 weeks of age and that the mRNA expression level of *Spp1* was positively correlated with the expression of *Fshb* in male rats. They also reported that *Spp1* plays an important role in cellular communication in gonadotropes, but is not regulated by GnRH. The results of all these studies suggest that changes in *Spp1* expression are closely related to FSH synthesis and secretion. With the aim of verifying this scientific hypothesis, this study confirmed that the protein OPN encoded by the *Spp1* gene colocalizes with FSHB and that *Spp1* could promote FSH synthesis and secretion by in vitro cellular experiments. The reduced expression of *Spp1* in the adult pituitary gland may be one of the molecular mechanisms that regulate the reduced level of FSH secretion in adulthood. However, the specific molecular mechanisms by which *Spp1* regulates FSH synthesis and secretion still need to be further explored. Several studies have confirmed that osteopontin (also known as SPP1, OPN), a protein encoded by the *Spp1* gene, can activate the MAPK signaling pathway to play a role in tumor progression [39], inflammatory responses [40], and neuroprotection [41]. Therefore, it is hypothesized that the *Spp1* gene or autocrine OPN may be involved in regulating pituitary function through the MAPK signaling pathway. This will also be part of our future work.

In addition, the pituitary *Spp1* gene, which is specifically enriched in gonadotropin cells, may not only function to regulate hormone synthesis. The *Spp1* gene is expressed in a wide range of tissues, and the SPP1protein acts as an exocrine protein whose paracrine and autocrine roles allow for the broader regulation of other glands in the body. It has been shown that macrophage-derived SPP1 has a protective effect against nonalcoholic steatohepatitis [42]. The TSH-SPP1/TRβ-TSH positive feedback loop has also been shown to be a participant in the regulation of fat deposition in the liver [43]. Besides the liver, SPP1 is considered to be both necessary and sufficient to induce hair growth [44]. The overexpression of SPP1 is associated with a poor prognosis of melanoma, and the inhibition of its expression suppresses the proliferation, migration, and invasion of melanoma cells [45]. A study by Gao Gong et al. on the comparative analysis of gene expression in skin tissues of different types and developmental stages of inner Mongolia cashmere goats also suggests that the differences in skin follicle development and hair type may also be closely related to the role of SPP1 [46]. Due to its biological functions related to immune regulation, the role of SPP1 in the progression of various tumors, including penile cancer [47], ovarian cancer [48], lung cancer [49], and intrahepatic cholangiocarcinoma [50], has been gradually discovered. More and more single-cell histological studies have shown that SPP1 has become a marker for the cellular typing of a variety of tumors. In the future, we also hope to explore more mechanisms in the potential role of autocrine SPP1 on the pituitary gland and the extra-pituitary role of paracrine SPP1.

In conclusion, this study constructed a single-cell transcriptome profile of the rat pituitary gland before and after sexual maturation. It was found that the expression of *Spp1*, which is specifically expressed in gonadotrope cells, decreased after sexual maturation, which in turn inhibited the synthesis and secretion of FSH. The results of this study provide a rich resource and data support for an in-depth investigation of the mechanisms of pituitary gland development and pituitary hormone synthesis and secretion.

## 4. Materials and Methods

### 4.1. Animals and Cells

SPF-grade male Sprague-Dawley (SD) rats at the ages of 4 weeks and 12 weeks were obtained from Liaoning Changsheng Biotechnology Co, Ltd. (Benxi, China). All animal experiments were reviewed by the Institutional Animal Care and Use Committee of Jilin University (Permit Number: SY202105051). The gonadotrope cell line LβT2 was kindly donated by Prof. Jing Liu from Zhejiang University, after receiving authorization from Prof. Pamela L Mellon. LβT2 cells were cultured in high-glucose DMEM containing 10% fetal bovine serum (FBS) and 1% penicillin–streptomycin and incubated in a humidified incubator with 5% CO_2_ at 37 °C.

### 4.2. Cell Suspension Preparation

Male SD rats were assigned to a pre-puberty group (aged 4 weeks) or a post-sexual maturity group (aged 12 weeks). Pituitary tissues were obtained from 4-week-old and 12-week-old rats (three rats in each group and mixed into one pool) after euthanasia. PBS-washed tissues were sheared and digested in DMEM high-glucose medium containing 0.25% collagenase type II (Invitrogen, Waltham, MA, USA), 0.125% trypsin (Invitrogen, Waltham, MA, USA), and 50 mg/mL DNase I (Roche, Manheim, Germany) at 37 °C for 60–90 min. Digestion was terminated by DMEM high-glucose medium containing 10% fetal bovine serum. The suspension was filtered through a 40 μm cell sieve and centrifuged at 200× *g* for 5 min. Single-cell suspensions with a concentration of approximately 700–1200 cells/μL were prepared by resuspending cells with Dulbecco’s phosphate-buffered saline (Sigma, St. Louis, MO, USA) containing 0.1% bovine serum albumin (Sigma, St. Louis, MO, USA). The cell number and viability of the single-cell suspensions were determined using a fluorescence cell counter (LUNA-FLTM, Anyang, Republic of Korea).

### 4.3. Cell Capture and RNA Library Construction

The fluorescence cell counter was used again to ensure the cell number and viability of the single-cell suspensions before the assay. Single-cell transcriptome 3′ cDNA libraries were constructed using a V3.1 kit for a 10× Genomics Chromium Controller (10× Genomics Inc., Pleasanton, CA, USA), according to the instructions, with an expected capture of 10,000 cells/passage. The cDNA libraries were qualified by a Qubit^®^ 3.0 Fluorometer (Life Technologies, Carlsbad, CA, USA) and an Agilent 2100 High Sensitivity DNA Assay Kit (Agilent, Santa Clara, CA, USA). Sequencing was performed on an Illumina NovaSeq 6000 PE150 platform (Illumina, San Diego, CA, USA) at Annoroad Gene Technology Co, Ltd. (Beijing, China).

### 4.4. Bioinformatics Analysis of scRNA-seq Data

The bioinformatics analysis of scRNA-seq data was performed in conjunction with published studies [51,52]. Cell Ranger Single Cell Software version 6.0.1 (10X Genomics, Inc.) was used to align and quantify sequenced raw reads against the reference genome (Rattus_norvegicus Rnor_6.0.89.chr). Background noise was removed and the filtered gene-barcode matrices only included cells with at least 300 UMI counts. Low-quality cells were removed based on a high mitochondrial content >20% (expressing gene “Mt-nd1”, “Mt-nd2”, “Mt-co1”, “Mt-co2”, “Mt-atp8”, “Mt-atp6”, “Mt-cox3”, “Mt-nd3”, “Mt-nd4l”, “Mt-nd4”, “Mt-nd5”, “Mt-nd6”, and “Mt-cyb”). A low number of detected genes <100 or a high number of detected genes >4000 were also filtered. Erythrocytes were removed based on cells expressing the genes “Hba-a3”, “Hba-a2”, ”Hba-a2.1”, and “Hbb”. 

A subsequent data analysis was carried out in the Seurat package (Seurat4.1.1) for normalization, identifying variable features, and optimizing modularity. UMAP dimensions were used to visualize the resulting clusters. Markers for each cluster were identified by meeting rules expressed in at least 20% of cells in the cluster and expressed at an at least log(0.25)-fold higher level in the cluster when compared with other clusters. Differentially expressed genes were analyzed (min.pct ≥ 0.10, |log2 fold change| ≥ 0.25, *p*_value ≤ 0.05, and q_value ≤ 0.05) and enrichment analyses of differentially expressed genes were taken by v4.6.0 R clusterProfiler (pad just < 0.05).

### 4.5. Cell Transfection

LβT2 cells were inoculated in six-well plates, and cell culture was performed by allowing the cell density to reach 40–60% confluency in DMEM high-glucose medium without FBS and penicillin–streptomycin. Plasmids (500 ng, 1000 ng, or 1500 ng) or siRNAs (100 nM) were transfected into LβT2 cells using the Lipofectamine™ 2000 transfection reagent (Thermo, Waltham, MA, USA) according to the manufacturer’s instructions. The medium was replaced with high-glucose DMEM containing 10% FBS after 6 h of transfection. mRNA expression changes were determined after 24 h and protein expression changes were determined after 48 h. All siRNAs were constructed by HippoBio (Huzhou, China), and the overexpression plasmid was constructed by GenePharma (Suzhou, China). The siRNA sequences used are detailed in Supplementary Table S1.

### 4.6. RT-qPCR

RNA from pituitary tissues and LβT2 cells was extracted by the SevenFast^®^ Total RNA Extraction Kit (Sevenbio, Beijing, China). The concentration and purity of RNA were determined by a NanoDrop ND-2000 spectrophotometer (NanoDrop Technologies, China). The MonScript™ 5× RTIII All-in-One Mix (Monad, Suzhou, China) was used for reverse transcription to obtain cDNA, and the MonAmp™ SYBR^®^ Green qPCR Mix (Monad, Suzhou, China) was used to determine mRNA expression by RT-qPCR, according to the manufacturer’s recommended protocol. Gapdh was used as the reference gene, and the 2^−∆∆Ct^ method was used to analyze the relative gene expression. The reverse transcription reaction system and program, RT-qPCR reaction system and program, and all primers used are detailed in Supplementary Table S2.

### 4.7. ELISA

Blood samples were collected from 4-week-old and 12-week-old male SD rats. The serum was obtained by centrifugation at 3000 rpm for 10 min at 4 °C after standing for 4 h at room temperature. Hormone levels of FSH (YX-E21136), LH (YX-E20754), GH (YX-E21088), PRL (YX-E21133), TSH (YX-E21145), and ACTH (YX-E21135) were determined using the corresponding rat ELISA kits (Sinobestbio, Shanghai, China), according to the manufacturer’s instructions. The levels of FSH in the culture supernatants of LβT2 cells were measured using the Mouse FSH ELISA Kit (ml001910, mlbio, Shanghai, China), according to the manufacturer’s instructions.

### 4.8. Immunofluorescence

Pituitary tissues were obtained from 4-week-old and 12-week-old male SD rats after euthanasia. After washing with ice-cold PBS, the tissues were fixed in paraformaldehyde fixative at 4 °C for 24 h. The fixed tissues were sliced into 5 μm paraffin sections for immunofluorescence. The sections were sequentially subjected to dehydration, antigen retrieval, blocking, multicolor immunostaining, counter staining for the nucleus, and mounting. Fluorescence microscopy was performed and images were acquired. The antibodies used were as follows: LHB (1:200, Cat# DF7140, Affinity, San Francisco, CA, USA); SPP1 (OPN, 1:200, Cat# 22952-1-AP, Proteintech, Rosemont, IL, USA); FSHB (1:1000, Cat# ab281562, Abcam, Cambridge, UK); and Cy3 (used for FSHB)-, Cy5 (used for LHB)-, or Alexa Fluor 488 (used for OPN)-labeled goat antirabbit IgG (1:400, SeraCare, Milford, MA, USA).

### 4.9. Data Analysis

The data are presented as the mean ± SD of three independent experiments. The data were analyzed using SPSS 19.0 software (SPSS, Chicago, IL, USA) and figures were generated using GraphPad Prism 9 (Prism for Mac OS X, GraphPad Software Inc., San Diego, CA, USA). *p* < 0.05 was considered to indicate statistical significance.

## Data Availability

The datasets during and/or analyzed during the current study are available from the corresponding author on reasonable request. The raw data have been uploaded to the NCBI BioProject. Accession for these NCBI BioProject data: PRJNA1084893.

## Supplementary Materials

The following supporting information can be downloaded at: https://www.mdpi.com/article/10.3390/ijms25094694/s1.

## Abbreviations

ACTH	Adrenocorticotropic hormone
ATP	Adeosine triphosphate
cAMP	Cyclic adenosine monophosphate
CREB	cAMP response element binding
FSH	Follicle-stimulating hormone
GH	growth hormone
GnRH	Gonadotropin-releasing hormone
LH	Luteinizing hormone
MAPK	Mitogen-activated protein kinase
NADPH	Nicotinamide adenine dinucleotide phosphate
PKA	Protein kinase A
PRL	Prolactin
SPP1	Secreted phosphoprotein 1
TRβ	Thyroid hormone receptor β
TSH	Thyroid stimulating hormone
