# Supplementary material for: Single-Cell Transcriptional Profile Construction of Rat Pituitary Glands before and after Sexual Maturation and Identification of Novel Marker Spp1 in Gonadotropes
Source: Int J Mol Sci. 2024 Apr 25;25(9):4694. doi: 10.3390/ijms25094694 (PMC11083676; doi:10.3390/ijms25094694)
Supplement: Supplementary file 1 [file ijms-25-04694-s001.zip › Supplementary Figures.pdf]

Single-cell transcriptional profile construction of rat pituitary glands before and after sexual maturation and identification of novel marker *Spp1* in gonadotropes

Qing-Hua Huang, Guo-Kun Zhao, Hao-Qi Wang, Fan-Hao Wei, Jin-Yu Zhang,
Jia-Bao Zhang, Fei Gao, Bao Yuan

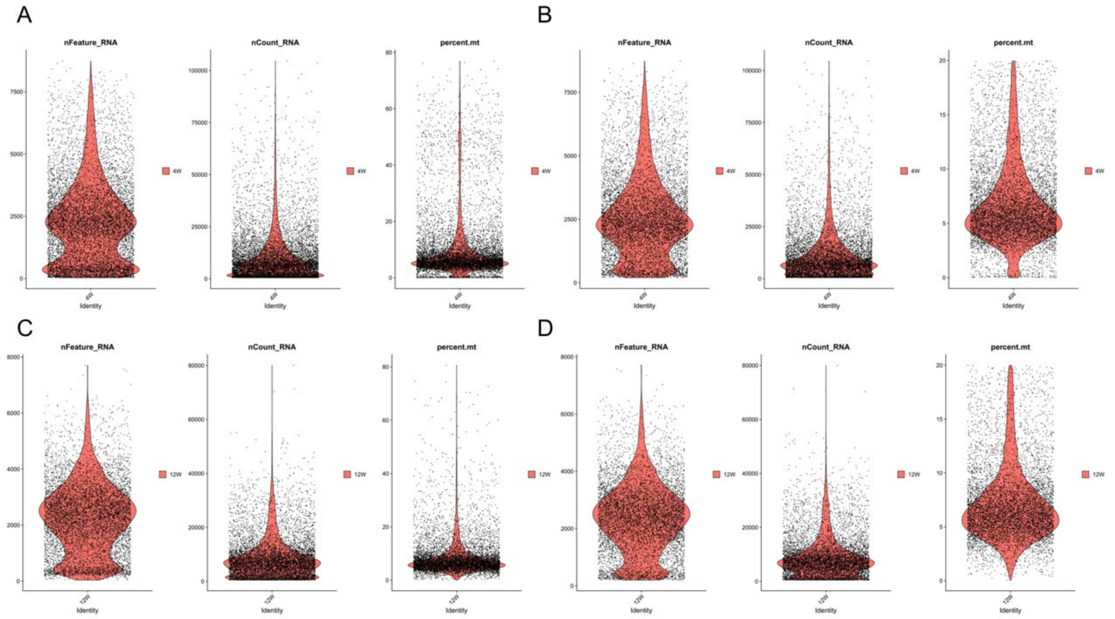

Figure S1. Violin plots of nFeature RNA, nCount RNA and mitochondria percent. (A, C) Violin plots of raw gene UMI mito percent. (B, D) Violin plots of filter gene UMI mito percent.

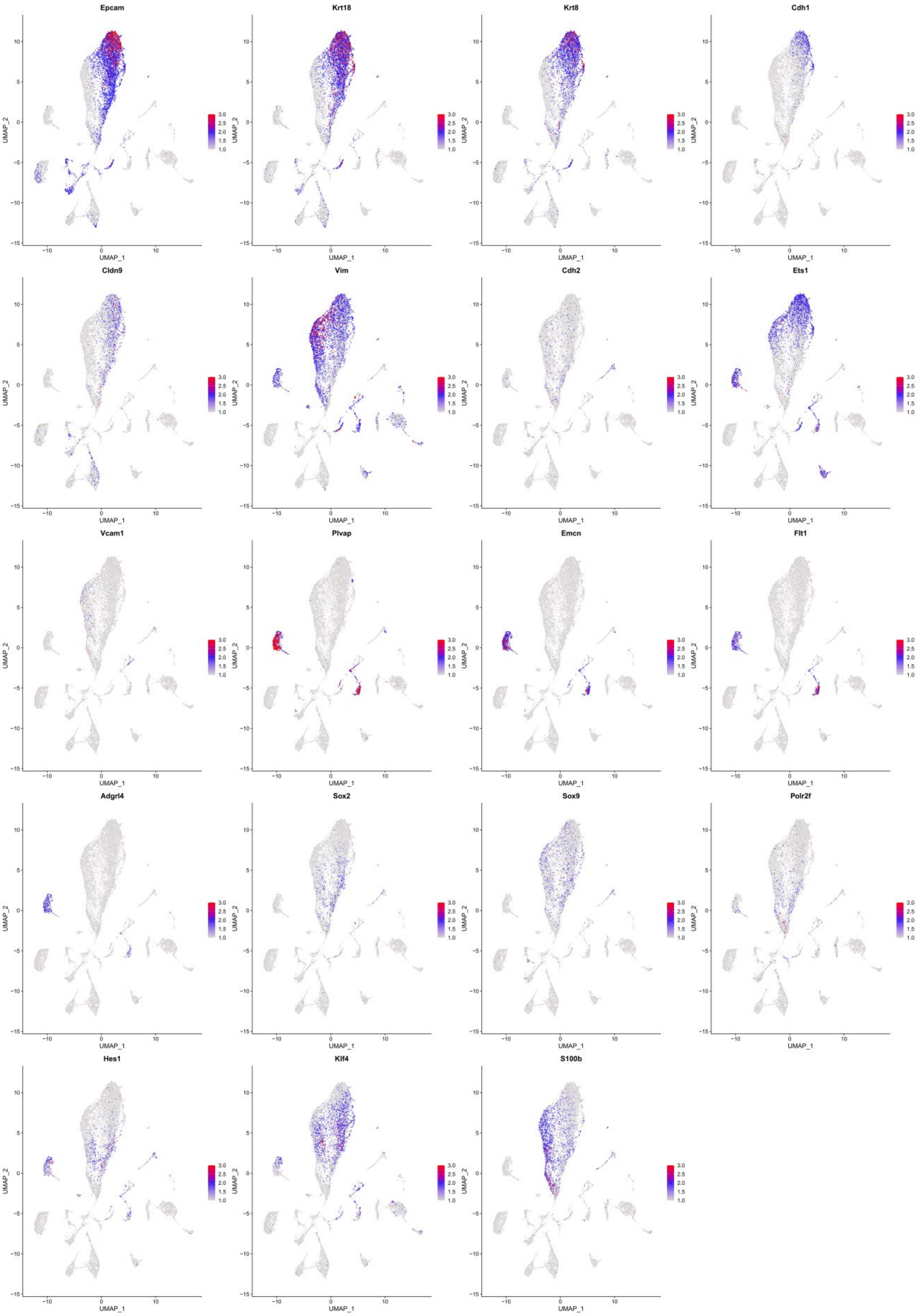

Figure S2. UMAP maps of marker genes in distinct pituitary non-hormone cell clusters.
